# Kentucky Giantess

**Published:** 1857-07

**Authors:** 


*Kentucky Giantess.*—There is now on exhibition in the Museum in N. Orleans, a Kentucky female, weighing 515 pounds, is nine feet two inches in circumference, and measures twenty-nine inches around the arm, and thirty-eight inches around the calf of the leg. Kentucky is rather remarkable for monsters in the human form.

